# Effects of ciprofol versus propofol sedation on hypoxaemia and hypotension in elderly patients undergoing bidirectional endoscopy: protocol for a randomized controlled trial

**DOI:** 10.3389/fmed.2026.1833766

**Published:** 2026-06-09

**Authors:** Yue Fei, Minjun Gu, Xinlong Li, Liangyu Zheng, Youjia Yu, Zhengjie Chen

**Affiliations:** 1 Department of Anesthesiology, Sir Run Run Shaw Hospital, School of Medicine, Zhejiang University, Hangzhou, Zhejiang, China; 2 Department of Anesthesiology, Xiangcheng People's Hospital, Suzhou, Jiangsu, China

**Keywords:** bidirectional endoscopy, ciprofol, elderly patients, hypotension, hypoxaemia, propofol

## Abstract

**Background:**

In elderly patients undergoing gastrointestinal endoscopy under sedation, respiratory and haemodynamic instability remains a significant concern. Compared with the commonly used propofol, ciprofol may offer improved cardiorespiratory stability. This study aims to evaluate the effects of ciprofol versus propofol on the incidence of hypoxaemia and hypotension in elderly patients undergoing bidirectional gastrointestinal endoscopy under sedation.

**Methods:**

This is a single-center, prospective, randomized, double-blind, parallel-controlled clinical trial conducted at a tertiary academic hospital. A total of 184 patients aged ≥65 years undergoing elective bidirectional gastrointestinal endoscopy under sedation will be randomly assigned in a 1:1 ratio to receive either ciprofol or propofol using block randomization stratified by hypertension status, with block sizes of 2 and 4. The primary outcome is the composite incidence of hypoxaemia (SpO₂ < 93%, for >10 s) and hypotension (systolic blood pressure <90 mmHg, mean arterial pressure <65 mmHg, or a ≥ 20% decrease from baseline) during sedation. Secondary outcomes will include the individual incidences of hypoxaemia and hypotension, airway interventions, use of vasoactive drugs, sedation and recovery times, total sedative dose, and patient and endoscopist satisfaction. Safety outcomes will include nausea or vomiting, dizziness, headache, respiratory depression, bradycardia and arrhythmias.

**Discussion:**

This study aims to provide high-quality evidence regarding whether ciprofol improves respiratory and haemodynamic safety compared with propofol in elderly patients undergoing bidirectional gastrointestinal endoscopy.

**Clinical trial registration:**

https://www.chictr.org.cn/showproj.html?proj=307374, ChiCTR2600117418.

## Introduction

Gastrointestinal diseases are highly prevalent among older adults, and gastrointestinal endoscopy has become an essential tool for screening, diagnosis, and therapeutic intervention (1). With the growing aging population, the number of endoscopic procedures continues to increase worldwide. Sedation during endoscopy is widely used to improve patient comfort and facilitate procedural performance. However, older patients often have reduced cardiopulmonary reserve and increased sensitivity to sedative agents, making sedation-related respiratory and haemodynamic instability a persistent clinical concern (2, 3).

Propofol remains the most commonly used sedative for gastrointestinal endoscopy because of its rapid onset, short duration of action, and favorable recovery profile (4). Nevertheless, propofol causes dose-dependent central nervous system depression and may impair respiratory and cardiovascular function (5). Previous studies have reported relatively high incidences of hypoxaemia and hypotension during propofol-based sedation in elderly patients undergoing endoscopic procedures, posing additional challenges for peri-procedural management (6).

Ciprofol is a novel intravenous sedative–hypnotic agent structurally derived from propofol. It acts on the *γ*-aminobutyric acid type A (GABA_A) receptor and has an estimated sedative potency approximately four to five times greater than propofol (7). Because effective sedation can be achieved at lower doses, ciprofol may be associated with reduced respiratory and haemodynamic suppression (8). Early clinical studies in endoscopic procedures suggest that ciprofol provides sedation efficacy comparable to propofol while potentially offering improved cardiorespiratory stability (9, 10).

Recent randomized trials and systematic reviews have shown that ciprofol provides sedative efficacy comparable to propofol in gastrointestinal endoscopy, with potential advantages in reducing injection pain, hypoxaemia, respiratory depression, and hypotension (11–15). However, whether ciprofol confers benefits in reducing respiratory and haemodynamic instability among elderly patients, a population particularly vulnerable to cardiorespiratory compromise, remains uncertain, as high-quality randomized controlled trial evidence is still lacking. The present trial aims to compare ciprofol with propofol in elderly patients undergoing bidirectional gastrointestinal endoscopy, using the composite incidence of hypoxaemia and hypotension as the primary outcome.

## Methods and analysis

This protocol follows the Standard Protocol Items: Recommendations for Interventional Trials (SPIRIT) guidelines.

### Study design and patients

**Figure 1:**
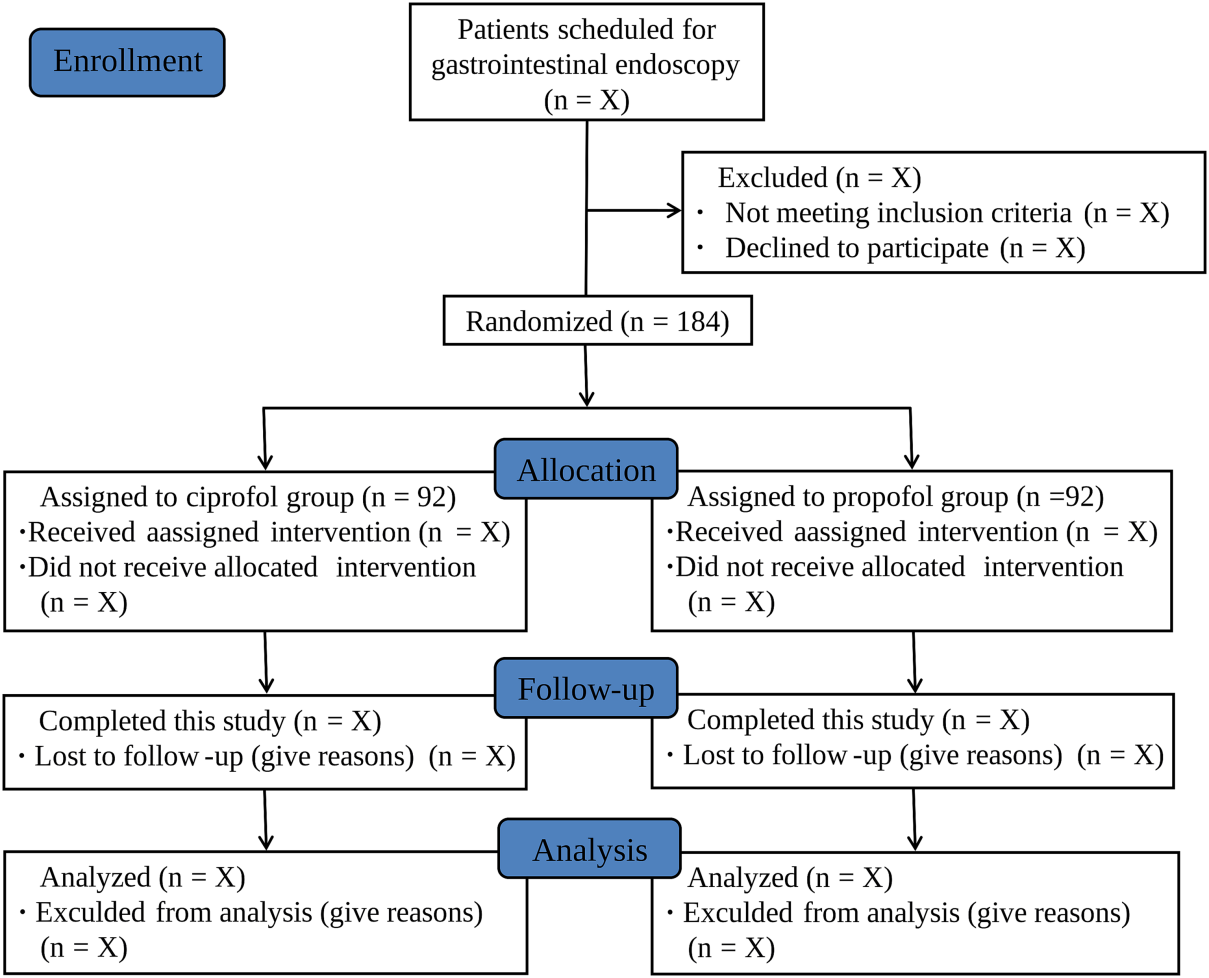
Study flowchart.

This study is a single-center, prospective, randomized, parallel-group clinical trial designed to compare the effects of ciprofol and propofol on the occurrence of hypoxaemia and hypotension during sedation in elderly patients undergoing gastrointestinal endoscopy. The study was approved by the Ethics Committee of Sir Run Run Shaw Hospital, Zhejiang University School of Medicine (approval no. 2026-093) and registered with the Chinese Clinical Trial Registry (ChiCTR2600117418). The trial will be conducted at Sir Run Run Shaw Hospital, Zhejiang University School of Medicine, enrolling a total of 184 patients. Recruitment is scheduled to take place between February 2026 and May 2026. The study flow diagram is shown in Figure 1.

### Inclusion criteria

- Age ≥ 65 years.
- Scheduled for elective gastrointestinal endoscopy and requires sedation.
- ASA physical status I-III.
- SpO₂ ≥ 93% under ambient air before the procedure.
- Understand the study content and voluntarily sign the informed consent form.

### Exclusion criteria

- Emergency endoscopy.
- Severe cardiopulmonary diseases (e.g., heart failure, COPD).
- SBP < 90 mmHg, MAP < 65 mmHg or HR < 50 bpm.
- Allergy to ciprofol, propofol, or opioids.
- Anticipated difficult airway.
- BMI > 30 kg/m^2^.
- Severe liver, kidney, neurological, or metabolic diseases.
- History of alcohol or drug abuse.

### Primary outcome

The primary outcome is the incidence of the composite outcome of hypoxaemia and hypotension during the sedation period. Hypoxaemia is defined as a decrease in peripheral oxygen saturation (SpO₂) to <93% for a duration exceeding 10 s. Hypotension is defined as systolic blood pressure <90 mmHg, mean arterial pressure <65 mmHg, or a decrease of ≥20% from baseline. For each participant, the composite outcome will be recorded only once regardless of the number of occurrences of any component event (Table 1).

**Table 1:** Schedule of patient enrolment, study interventions and outcome assessment.

Time point	Study period				
	Enrolment	Allocation	Post-allocation		Close-out
	Preanesthetic visit	60 min before sedation	During procedure	End of procedure	PACU discharge
Patient enrolment					
Eligibility criteria	×				
Written informed consent	×				
Demographic data	×				
Baseline characteristics	×				
Randomization		×			
Allocation		×			
Study interventions					
Ciprofol			×		
Propofol			×		
Outcome assessment					
Hypotension			×		
Hypoxemia			×		
Use of vasopressors			×		
Sedation induction time			×		
Time to full awakening (MOAA/S = 5)					×
PACU stay time					×
Total sedation drug dose				×	
Patient satisfaction					×
Endoscopist satisfaction				×	
Adverse events					
Nausea and vomiting					×
Headache					×
Dizziness					×
Respiratory depression			×	×	×
Apnea			×	×	×
Bradycardia			×	×	×
Arrhythmia			×	×	×

### Secondary outcomes

Secondary outcomes include the individual incidences of hypoxaemia and hypotension; airway interventions (e.g., mandibular lift, oral or nasal airway placement, or assisted ventilation); use of vasoactive drugs; time to achieve intravenous induction; time to full alertness (defined as a Modified Observer’s Assessment of Alertness/Sedation [MOAA/S] score of 5); duration of stay in the post-anesthesia care unit (PACU); total dose of sedative medications; patient satisfaction assessed using a 10-point visual analogue scale (VAS); and endoscopist satisfaction assessed using a 10-point VAS.

### Safety outcomes

Safety outcomes will include the incidence of adverse events, including nausea and vomiting, dizziness, headache, respiratory depression, apnea, bradycardia, and arrhythmias. Respiratory depression will be defined as a respiratory rate <8 breaths/min or the need for airway intervention. Apnea will be defined as absence of spontaneous breathing for >15 s. Bradycardia will be defined as heart rate <50 beats/min. Arrhythmia will be defined as any new-onset rhythm abnormality detected by electrocardiographic monitoring. Other adverse events will be recorded when reported by the participant or observed by the investigator.

### Randomization and blinding

Randomization will be performed using an online randomization tool (https://www.sealedenvelope.com/simple-randomiser/v1/lists) with a 1:1 allocation ratio. Eligible participants will be assigned to either the ciprofol group or the propofol group. Stratified block randomization will be applied according to the presence of hypertension, with random block sizes of 2 and 4. The randomization sequence will be generated by an independent investigator not involved in patient recruitment, intervention delivery, or outcome assessment. Allocation concealment will be ensured using sealed, opaque, sequentially numbered envelopes prepared in advance.

Because both ciprofol and propofol are milky white lipid emulsions with identical appearance, participants, anesthesiologists responsible for sedation, perioperative care providers, outcome assessors, and statisticians will remain blinded to treatment allocation throughout the study.

### Anesthetic care

All participants will fast for at least 8 h and abstained from drinking for at least 4 h before the procedure. Upon arrival in the gastrointestinal suite, patients will receive monitoring of electrocardiography, pulse oximetry, respiratory rate and non-invasive cuff blood pressure on the right upper arm at 2-min intervals. Supplemental oxygen will be continuously administered via nasal cannula at a flow rate of 5 L/min. All patients will receive intravenous fentanyl at a dose of 1 μg/kg prior to sedation induction.

Participants will then receive the allocated sedative agent according to group assignment. Ciprofol will be supplied at a concentration of 2.5 mg/mL and propofol at a concentration of 10 mg/mL. In the ciprofol group, ciprofol will be administered intravenously at an induction dose of 0.2–0.3 mg/kg over no less than 30 s, with supplemental boluses of 0.05 mg/kg administered as needed to maintain adequate sedation. In the propofol group, propofol will be administered intravenously at an induction dose of 1.0–1.5 mg/kg over no less than 30 s, with supplemental boluses of 0.25 mg/kg administered as needed. Accordingly, the corresponding induction injection volumes will be 0.08–0.12 mL/kg for ciprofol and 0.10–0.15 mL/kg for propofol, with substantially overlapping volume ranges. Sedation will be administered according to the same standardized protocol in both groups, with supplemental dosing guided by predefined sedation criteria and patient responses.

The target sedation depth will be defined as MOAA/S ≤ 1 (16) during upper gastrointestinal endoscopy and MOAA/S ≤ 2 during colonoscopy and will be assessed at 5-min intervals throughout the bidirectional endoscopic procedure, with sedative doses adjusted according to patient responsiveness and vital signs. Bidirectional endoscopy will be defined as same-session upper gastrointestinal endoscopy and colonoscopy performed in the same participant. In all cases, upper gastrointestinal endoscopy will be performed first, followed by colonoscopy.

Airway management will follow a predefined stepwise protocol and will be initiated in cases of respiratory depression, apnoea, suspected airway obstruction, clinically inadequate ventilation, or hypoxaemia. Interventions will include jaw thrust, airway adjuncts such as a nasopharyngeal airway when necessary, and escalation to advanced airway management, such as tracheal intubation, if adequate oxygenation or ventilation cannot be maintained. Hypotension will be treated with intravenous ephedrine 6 mg or phenylephrine 50 μg. Bradycardia will be treated with intravenous atropine 0.25 mg.

At the end of the procedure, patients will be transferred to PACU. PACU discharge will be permitted when the Modified Aldrete Score is ≥9; patients not meeting this criterion will be reassessed every 2 min until discharge. Patient satisfaction using a 10-point VAS will be assessed before PACU discharge after recovery, while endoscopist satisfaction will be assessed immediately after the procedure. All endoscopic procedures will be conducted by a consistent team of experienced gastroenterologists and anesthesia providers according to standardized institutional protocols.

### Data collection and monitoring

Baseline demographic and clinical data, including age, sex, body mass index (BMI), American Society of Anesthesiologists (ASA) physical status, chronic obstructive pulmonary disease (COPD), asthma, bronchiectasis, obstructive sleep apnea (OSA), hypertension, diabetes, stroke, malignancy, smoking status, alcohol consumption, education level, number of previous painless endoscopies, and Mallampati score, will be collected before the procedure.

Primary and secondary outcomes, as well as other periprocedural data, will be recorded during the sedation and recovery periods by investigators blinded to treatment allocation. All data will be documented on standardized case report forms (CRFs) and entered into a secure electronic database. According to the prespecified statistical analysis plan, statistical analyses will be performed by an independent statistician using a de-identified dataset. An independent Data and Safety Monitoring Committee (DSMC) will oversee the trial and conduct ongoing reviews of trial implementation and safety.

### Sample size calculation

Hypoxaemia and hypotension are commonly reported separately in previous studies of propofol-based endoscopic sedation in various patient populations. In this study, these two clinically relevant events were combined as a composite primary outcome to better capture sedation-related cardiopulmonary instability in elderly patients. Based on pilot observations and previously published studies (17, 18), the incidence of the primary composite outcome in the propofol group was estimated to be approximately 35%, while the expected incidence in the ciprofol group was estimated to be 16%. We hypothesized that ciprofol would reduce this incidence to 16%, corresponding to an absolute risk reduction of 19 percentage points. Assuming a two-sided *α* level of 0.05, 80% statistical power, and a 1:1 allocation ratio, a sample size of 82 participants per group was calculated using a two-sample comparison of proportions. Allowing for an anticipated dropout or loss-to-follow-up rate of approximately 10%, the final target sample size is 184 participants, with 92 participants in each group.

### Statistical analysis

Normality of continuous variables will be assessed using the Shapiro–Wilk test. Normally distributed variables will be presented as means ± standard deviation (SD), and group comparisons will be performed using the independent *t*-test. Non-normally distributed variables will be expressed as medians with interquartile ranges (IQR), with group comparisons conducted using the Mann–Whitney U test. Categorical variables will be expressed as frequencies and percentages, and comparisons between groups will be made using the chi-square test (χ^2^) or Fisher’s exact test, depending on the expected frequencies.

The balance of baseline characteristics between groups will be evaluated using standardized mean differences (SMD), which quantifies potential imbalances following randomization. Treatment effects will be reported as odds ratios or mean differences (MD) with 95% confidence intervals.

The primary outcome (a composite of hypoxemia and hypotension) will be compared between groups using the chi-square test, followed by multivariable logistic regression analysis to adjust for pre-specified covariates, including age, BMI, smoking status, hypertension and diabetes. Additional models will be performed to adjust for baseline variables with an SMD > 0.10, as well as a model adjusting only for hypertension and age, to assess robustness.

Pre-specified subgroup analyses of the primary outcome will be performed according to age (≤ 75 vs. > 75 years), smoking status, hypertension, and diabetes. Interaction tests will be conducted to explore potential heterogeneity of treatment effects.

Data will primarily be analyzed in the intention-to-treat (ITT) population, which includes all randomized patients who underwent the endoscopic procedure and have available primary outcome data. A per-protocol (PP) analysis will also be performed as a sensitivity analysis. No interim analyses will be conducted, and missing data for the primary outcome will not be imputed.

All statistical tests will be two-sided, with *p* < 0.05 considered statistically significant. Analyses will be performed using SPSS software (version 25.0; IBM, United States).

## Discussion

This randomized, double-blind, parallel-group clinical trial involves elderly patients undergoing gastrointestinal endoscopy and aims to compare the cardiorespiratory safety of ciprofol and propofol during procedural sedation. The primary objective is to evaluate whether ciprofol reduces the incidence of sedation-related cardiopulmonary instability, defined as the composite outcome of hypoxaemia and hypotension during the sedation period. Secondary objectives include evaluating other aspects of sedation performance and recovery, such as the individual incidences of hypoxaemia and hypotension, airway interventions, use of vasoactive drugs, time to full alertness, recovery time, total sedative consumption, and satisfaction ratings from both patients and endoscopists. This study will be conducted and reported in accordance with the Consolidated Standards of Reporting Trials (CONSORT) guidelines. These methodological considerations are intended to ensure the validity, transparency, and reliability of the study findings.

Sedation-related respiratory or haemodynamic instability remains a key safety concern during gastrointestinal endoscopy in elderly patients (19). Previous studies have evaluated the effects of ciprofol on respiratory or haemodynamic stability across different populations and using various comparators (16, 20, 21). In contrast, the present study focuses specifically on elderly patients with increased cardiopulmonary vulnerability, in whom cardiopulmonary adverse events represent clinically important outcomes that reflect the safety of intravenous sedation. Therefore, this study will use the composite incidence of hypoxaemia and hypotension as the primary outcome to comprehensively assess sedation-related respiratory and haemodynamic instability.

The potential differences in cardiorespiratory safety between ciprofol and propofol may be partly explained by their pharmacodynamic characteristics. Although both agents exert their hypnotic effects through potentiation of *γ*-aminobutyric acid type A (GABA_A) receptor–mediated inhibitory neurotransmission, ciprofol demonstrates higher receptor affinity and greater sedative potency (22). As a consequence, adequate sedation may be achieved at lower plasma concentrations compared with propofol (23). Because respiratory depression and haemodynamic instability during procedural sedation are often concentration dependent, reduced systemic drug exposure may translate into improved physiological stability, particularly in elderly patients with limited cardiopulmonary reserve.

Another potential explanation relates to the differential impact of sedative agents on ventilatory control and autonomic regulation. Sedative-hypnotic drugs can attenuate the central respiratory drive and blunt reflex responses to hypoxia and hypercapnia (24). In elderly patients, these protective reflexes are already diminished due to age-related changes in respiratory mechanics and neural control (25). A sedative regimen requiring lower effective concentrations may therefore exert less suppression of ventilatory responsiveness, potentially reducing the risk of hypoxaemia during endoscopic procedures.

Haemodynamic effects may also contribute to differences in safety profiles between sedative agents. Propofol is known to reduce systemic vascular resistance and myocardial contractility through both central sympathetic inhibition and direct vascular smooth muscle relaxation (26). These effects can predispose patients to hypotension, particularly in older individuals with impaired cardiovascular compensatory mechanisms (27). If effective sedation can be achieved with lower pharmacological exposure, the magnitude of these haemodynamic effects may be attenuated, which may help explain the improved haemodynamic stability observed in some studies of ciprofol.

Although several previous studies have compared ciprofol with propofol in endoscopic sedation, most have primarily evaluated sedation success rates or individual adverse events (16, 21). Their effects on cardiorespiratory stability in elderly patients, a population particularly vulnerable to sedation-related complications have been less well studied. Consequently, the overall burden of cardiopulmonary instability during procedural sedation has not been fully characterized. By using a composite safety endpoint, the present study provides additional insight into the comparative cardiorespiratory safety profiles of these two sedative agents in elderly patients. Nevertheless, cardiopulmonary safety during endoscopic sedation is not determined by the sedative agent alone. Increasing attention has been paid to the optimization of sedation strategies, and adjunctive medications may also influence the occurrence of hypoxaemia and hypotension. For example, recent work on lidocaine-assisted intravenous sedation suggests that ciprofol combined with lidocaine may improve respiratory and haemodynamic tolerance during endoscopic procedures (28).

## Limitations

This study has several limitations. First, as the trial is planned to be conducted at a single tertiary center, clinical practice patterns in other endoscopy settings may differ; therefore, the generalizability of the future findings should be interpreted in the context of local practice environments. Second, although the sample size has been calculated based on the composite primary endpoint, the study may not be sufficiently powered to detect small differences in individual adverse events. Third, follow-up is planned to be limited to the peri-procedural and in-hospital period; therefore, delayed adverse events occurring after discharge may not be fully captured.

## Conclusion

The primary aim of this randomized, double-blind clinical trial is to compare the cardiorespiratory safety of ciprofol and propofol during sedation in elderly patients undergoing gastrointestinal endoscopy. By focusing on the composite outcome of hypoxaemia and hypotension, the study seeks to determine whether ciprofol-based sedation may provide improved respiratory and haemodynamic stability compared with propofol in this high-risk population. The findings are expected to provide clinically relevant evidence to inform sedative selection and optimize peri-procedural sedation strategies for elderly patients undergoing gastrointestinal endoscopic procedures.
